# DECREASING THE USE OF BENZODIAZEPINES AND SEDATIVE HYPNOTICS IN POST OPERATIVE OLDER ADULTS

**DOI:** 10.1093/geroni/igac059.3053

**Published:** 2022-12-20

**Authors:** Amaka Opute

**Affiliations:** UTSW Medical Center Dallas, Dallas, Texas, United States

## Abstract

**Purpose:**

The purpose of this project was to decrease the prescribing of benzodiazepines and sedative hypnotics in post-operative older adults.

**Background:**

Routine prescribing of benzodiazepines and sedative hypnotics as first line choices for sleep medication is a known contributor to prolonged hospital stays, increased fall rates, increased development of delirium, higher health care costs, and long-term cognitive decline. Methodology: Practice changes included monthly provider and nursing staff education inservices, coupled with transparent monitoring of provider prescribing practices. The outcomes measure was to decrease the prescribing of benzodiazepines and sedative hypnotics by 5% within 90 days and increase the prescribing of melatonin by 10%.

**Results:**

When comparing six months of data pre and post implementation on the unit, there was a 33% decrease in the prescribing of benzodiazepines/sedative hypnotics and a 12% increase in the prescribing of Melatonin. There was also a 28% decrease in the hours of patient safety sitter use, a marker for delirium. Implications: This quality improvement project decreased the number of benzodiazepines and sedative-hypnotics prescribed by expanding provider and nursing knowledge, which ultimately led to fewer hours spent utilizing patient safety sitters, thereby lowering healthcare costs, while concurrently improving the quality of care delivered

